# Baseline Immune Signatures in Serum Extracellular Vesicles Distinguish Food-Induced from Wheat-Dependent Exercise-Induced Anaphylaxis

**DOI:** 10.3390/ijms27114732

**Published:** 2026-05-25

**Authors:** Junda Li, Tengze Shang, Kai Guan, Jia Yin

**Affiliations:** 1 Department of Allergy, Peking Union Medical College Hospital, Chinese Academy of Medical Sciences, Peking Union Medical College, Beijing 100730, China; lijunda0701@163.com (J.L.); shangtengze@163.com (T.S.); 2 Beijing Key Laboratory of Precision Medicine for Diagnosis and Treatment on Allergic Diseases, Peking Union Medical College Hospital, Beijing 100730, China; 3 National Clinical Research Center for Dermatologic and Immunologic Disease, Peking Union Medical College Hospital, Beijing 100730, China

**Keywords:** extracellular vesicles, food-induced anaphylaxis, wheat-dependent exercise-induced anaphylaxis (WDEIA), proteomics, biomarker

## Abstract

Food-induced anaphylaxis (FIA) is a life-threatening allergic reaction, while wheat-dependent exercise-induced anaphylaxis (WDEIA) is triggered by wheat ingestion plus cofactors. To elucidate their differences, we profiled serum extracellular vesicle (EV) proteomes from 240 participants, including WDEIA, FIA, oral allergy syndrome (OAS), and healthy controls. All blood samples were obtained at least one month after the most recent acute allergic reaction, using TMT-based LC-MS/MS with ELISA validation. A total of 583 EV proteins were confidently identified, revealing distinct immune features. Compared with controls, EV-derived C1-inhibitor (C1-INH) significantly decreased in both WDEIA and FIA, showing diagnostic potential for systemic anaphylaxis. Seventy-six proteins differed between WDEIA and FIA, with reduced apolipoprotein E (APOE) in FIA and elevated eosinophil cationic protein (ECP) in WDEIA, both exhibiting good discriminatory power. These findings indicate that serum EV proteomics can reveal unique immune signatures and identify C1-INH, APOE, and ECP as potential biomarkers distinguishing food-related anaphylaxis subtypes.

## 1. Introduction

Immunoglobulin E (IgE)-mediated food allergy affects approximately 10% of the global population, posing a significant burden on patients, families, and healthcare systems worldwide [1]. Food-induced anaphylaxis (FIA) is a rapid-onset, potentially life-threatening systemic allergic reaction that occurs upon exposure to specific food allergens [2]. Despite advances in allergy management and patient education, the unpredictable nature of anaphylaxis continues to contribute significantly to morbidity and mortality [3,4].

In China, wheat has emerged as a predominant cause of food-induced anaphylaxis, particularly in adults [5,6]. A clinical study involving 907 patients and 1952 episodes of anaphylaxis found that wheat was responsible for 20% of food-induced anaphylaxis cases in adolescents and 42% in adults [7]. Wheat-dependent exercise-induced anaphylaxis (WDEIA) is a distinct subtype of food-induced anaphylaxis that occurs specifically when wheat ingestion is followed by physical exercise, with other cofactors such as stress, alcohol intake, or nonsteroidal anti-inflammatory drugs (NSAIDs) also serving as precipitating factors [8]. In contrast, classical FIA is typically triggered by food exposure alone and does not require such cofactors, underscoring differences in triggering mechanisms despite overlapping clinical manifestations. In the Chinese population, apart from WDEIA, FIA is predominantly caused by fruits and vegetables, differing from the allergen profiles commonly reported in Western countries [7]. To date, systematic comparative studies investigating the molecular differences between WDEIA and classical FIA remain limited, highlighting the need to improve understanding of their underlying mechanisms, develop subtype-specific diagnostic tools, and advance personalized management strategies.

Extracellular vesicles (EVs)—nanoscale, lipid bilayer-bound vesicles secreted by nearly all cell types—have recently garnered attention as important mediators of immune regulation and disease pathogenesis [9]. EVs circulate in human biofluids and encapsulate a diverse array of molecular cargos, including proteins, RNAs (including microRNAs), and lipids, thereby serving as messengers of intercellular communication [10,11,12,13]. Increasing evidence supports their role in inflammatory and immune-mediated diseases, including allergic conditions [14,15,16].

Advances in high-throughput omics technologies, particularly proteomics, have enabled the systematic profiling of EV-associated proteins in various diseases [17,18]. In particular, tandem mass tag (TMT)-based quantitative mass spectrometry, offers a robust platform for uncovering differential protein expression patterns of small samples that may underline disease heterogeneity and guide biomarker development [19]. In this study, we performed comprehensive proteomic analyses of serum-derived EVs to investigate the molecular diversity underlying WDEIA and FIA, with the aim of identifying potential diagnostic biomarkers capable of distinguishing between WDEIA and FIA, as well as differentiating anaphylaxis from oral allergy syndrome (OAS), and to further elucidate their distinct immunological profiles.

## 2. Results

### 2.1. Validation of Serum-Derived EVs Isolation from Serum

**Figure 1:**
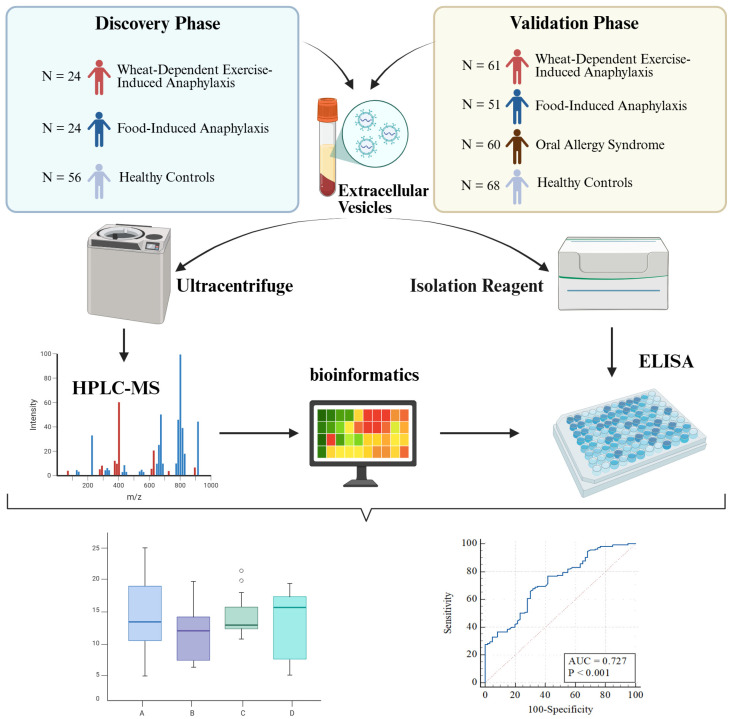
Schematic overview of the study design.

**Figure 2:**
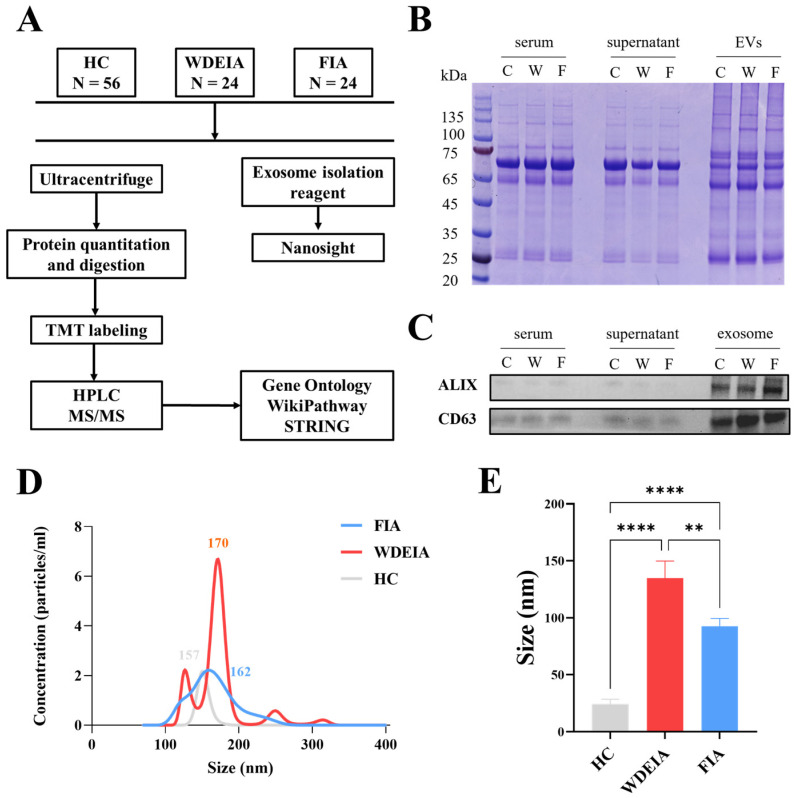
Schematic workflow and characterization of extracellular vesicles (EVs) isolated from pooled serum samples. (**A**) Schematic diagram of the TMT-based quantitative proteomic workflow for EVs isolated from pooled serum. (**B**) Coomassie-stained SDS-PAGE gel showing the total protein profiles and (**C**) Western blot analysis of EVs-specific markers ALIX and CD63 in harvested proteins of pooled serum samples. C: healthy control (a pool of 56 samples); W: WDEIA (a pool of 24 samples); F: FIA (a pool of 24 samples). (**D**) Particle size distribution profiles of serum EVs in the three groups as determined by nanoparticle tracking analysis. (**E**) Comparison of mean particle diameters among groups. “HC” refers to healthy control”; “WDEIA” refers to wheat-dependent exercise-induced anaphylaxis; “FIA” refers to food-induced anaphylaxis. Data are presented as mean ± SEM, one-way analysis of variance (ANOVA) followed by Tukey’s post hoc test to compare multiple groups, ** *p* < 0.01, **** *p* < 0.0001.

A schematic overview of the study design is shown in Figure 1. In discovery phase, Serum-derived EVs were isolated from healthy controls, WDEIA patients, and FIA patients (workflow shown in Figure 2A). Protein profiles of the resulting vesicle preparations were assessed by SDS-PAGE (Figure 2B), which revealed highly consistent banding patterns across all three groups. Western blot analysis demonstrated strong enrichment of the canonical EV markers ALIX and CD63 within the EV fractions (Figure 2C), both proteins were detected in the vesicle preparations and were markedly elevated compared to whole serum and the post-ultracentrifugation supernatants. Vesicle size distribution and concentration were then characterized by Nanoparticle tracking analysis (NTA) (Figure 2D). Both parameters were significantly elevated in WDEIA patients compared to FIA patients and healthy controls. In addition, FIA patients also exhibited higher EV size and concentration than healthy controls (Figure 2E and Figure S1A).

Labeled peptides derived from serum EVs of WDEIA patients, FIA patients, and healthy controls were analyzed using TMT-based quantitative mass spectrometry, with peptides from all groups pooled and measured together in a single batch to minimize batch effects. Applying stringent identification criteria (≥2 unique peptides per protein and FDR ≤ 0.01), the total of 583 proteins were identified with high confidence. Of these, 485 proteins (83.2%) were catalogued in the ExoCarta database (http://www.exocarta.org (accessed on 21 October 2025), Figure S1B). GO enrichment analysis revealed that most identified proteins localize to the collagen-containing extracellular matrix, enriched molecular functions included cadherin binding, antigen binding, and glycosaminoglycan binding, while enriched biological processes encompassed wound healing, regulation of body fluid levels, and blood coagulation (Figure S1C–E). These functional categories align with previously reported roles of EVs [20].

### 2.2. WDEIA- and FIA-Derived Serum EVs Present Similar Differentially Expressed Proteins

**Figure 3:**
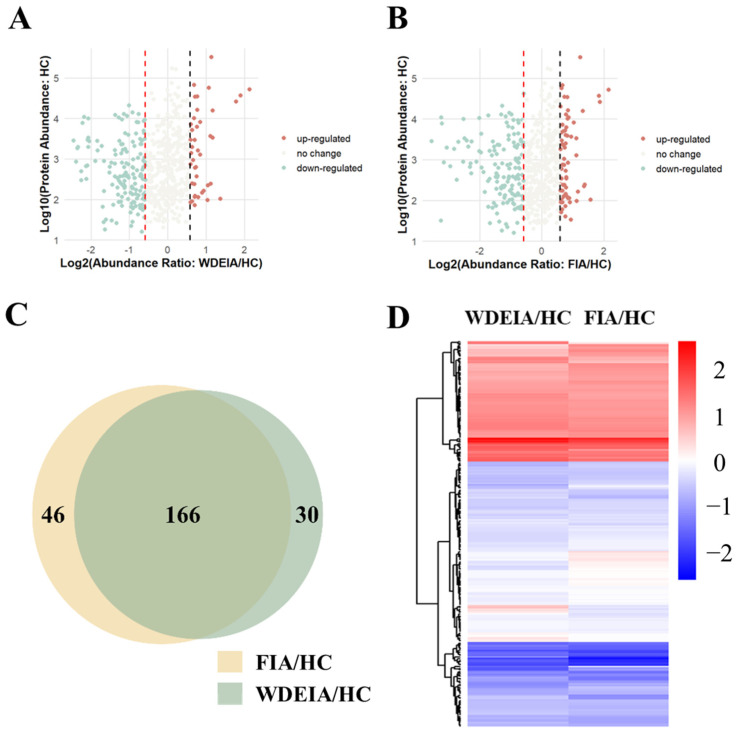
Comparative proteomic analysis of serum extracellular vesicles (EVs) from healthy controls, WDEIA, and FIA patients. (**A**,**B**) Scatter plots illustrate the distribution of differentially expressed proteins (DEPs) in WDEIA (**A**) and FIA (**B**) relative to healthy controls. Red dots indicate upregulated proteins; green dots indicate downregulated proteins. (**C**) Venn diagram of DEPs in WDEIA and FIA compared to healthy controls. (**D**) Heatmap of DEPs identified in WDEIA and FIA compared to healthy controls, showing hierarchical clustering of expression patterns. Red indicates upregulation, and blue indicates downregulation. “HC” refers to healthy control”; “WDEIA” refers to wheat-dependent exercise-induced anaphylaxis; “FIA” refers to food-induced anaphylaxis.

Differentially expressed proteins (DEPs) were defined by fold changes ≥ 1.50 (upregulated) or ≤0.67 (downregulated). Compared to healthy controls, 196 DEPs were identified in the WDEIA group, including 43 upregulated and 153 downregulated proteins (Figure 3A). In the FIA group, 212 DEPs were identified, with 67 upregulated and 145 downregulated (Figure 3B). Among these, 166 DEPs were shared between the WDEIA and FIA groups (Figure 3C).

To further characterize the expression patterns of DEPs in WDEIA and FIA, hierarchical clustering analysis was performed, and a heatmap was generated (Figure 3D). The results revealed a similar protein expression profile of DEPs between the two groups. Correlation analysis further confirmed a strong concordance in DEPs expression levels between WDEIA and FIA patients (r = 0.963, *p* < 0.001, Figure S2A). KEGG pathway enrichment analysis showed that these DEPs were primarily involved in complement and coagulation cascade pathway (Figure S2B).

### 2.3. Significant Reduction in C1-Inhibitor in Serum-Derived EVs from WDEIA and FIA Patients

**Figure 4:**
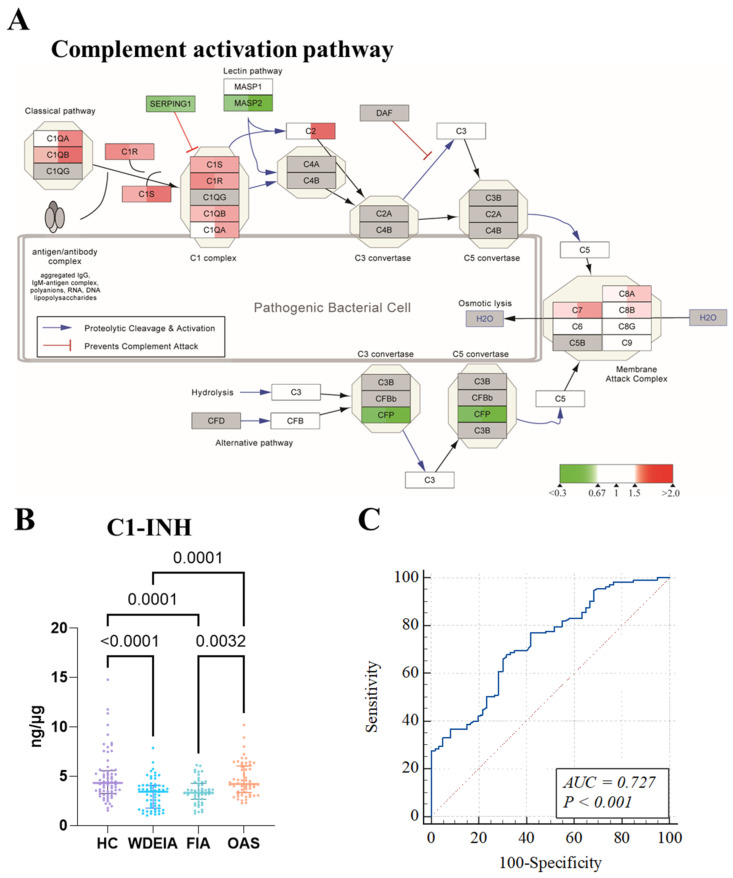
Pathway enrichment analysis of shared differentially expressed proteins (DEPs) in WDEIA and FIA, and validation of extracellular vesicles (EVs)-derived C1-inhibitor (C1-INH) levels. (**A**) Differentially expressed proteins (DEPs) shared between WDEIA and FIA groups mapped to the complement activation pathway. The comparison of WDEIA versus healthy controls is displayed on the left, and FIA versus healthy controls is shown on the right. Upregulated proteins are shown in red and downregulated proteins in green. Left indicates fold changes in WDEIA vs. healthy controls; right indicates fold changes in FIA vs. healthy controls. (**B**) EVs-derived C1-INH levels in healthy controls (*n* = 68), WDEIA (*n* = 61), FIA (*n* = 51), and oral allergy syndrome (OAS) (*n* = 60) groups, measured by ELISA. (**C**) Receiver operating characteristic (ROC) curve analysis evaluating the diagnostic performance of EVs-derived C1-INH for distinguishing systemic anaphylaxis (WDEIA and FIA) from OAS. “HC” refers to healthy control”; “WDEIA” refers to wheat-dependent exercise-induced anaphylaxis; “FIA” refers to food-induced anaphylaxis. Data are presented as mean ± SEM, one-way analysis of variance (ANOVA) followed by Tukey’s post hoc test to compare multiple groups.

To further elucidate the biological pathways involved in anaphylaxis, the WEB-based Gene SeT AnaLysis Toolkit was utilized to analyze the WikiPathways database. Eighteen proteins were enriched in the complement activation pathway. Notably, the majority of DEPs associated with this pathway, including C1QB, C1R, C1S, C7, and C8A, were upregulated in both WDEIA and FIA patients. In contrast, SERPING1, which encodes C1-Inhibitor (C1-INH), was significantly downregulated in serum-derived EVs from both patient groups (Figure 4A). These results suggest that EV-associated complement system activation may play a role in the pathophysiology of anaphylaxis.

Given the critical regulatory function of C1-INH in the complement cascade, we performed ELISA to validate the proteomic findings by quantifying C1-INH levels in serum-derived EVs from individual subjects. Consistent with the mass spectrometry results, ELISA confirmed that C1-INH levels were significantly lower in WDEIA and FIA patients compared to healthy controls. Furthermore, C1-INH levels were also significantly reduced in WDEIA and FIA patients compared to those with OAS (Figure 4B). To assess the diagnostic utility of EV-derived C1-INH in distinguishing anaphylaxis from OAS, ROC curve analysis was conducted. The area under the curve (AUC) was 0.727 (95% confidence interval [CI]: 0.654–0.792), indicating moderate diagnostic accuracy. At the optimal cutoff value of 3.73 ng/μg (sensitivity 67.86%, specificity 68.33%), C1-INH demonstrated good predictive ability for identifying anaphylaxis (Figure 4C).

### 2.4. APOE and ECP as Potential Diagnostic Biomarkers for WDEIA

According to the mass spectrometry analysis, 76 non-overlapping DEPs were identified between the WDEIA and FIA groups. GO enrichment analysis revealed that these DEPs were primarily involved in humoral immune responses and leukocyte-mediated immunity (Figure S3). Protein–protein interaction networks of these DEPs were constructed using Cytoscape based on STRING scores, revealing an intricate interaction network (Figure S4). Among these, APOE and ECP were identified as key hub proteins distinguishing WDEIA from FIA, as APOE showed the most significant downregulation in the FIA group, while ECP exhibited the greatest upregulation in the WDEIA group.

**Figure 5:**
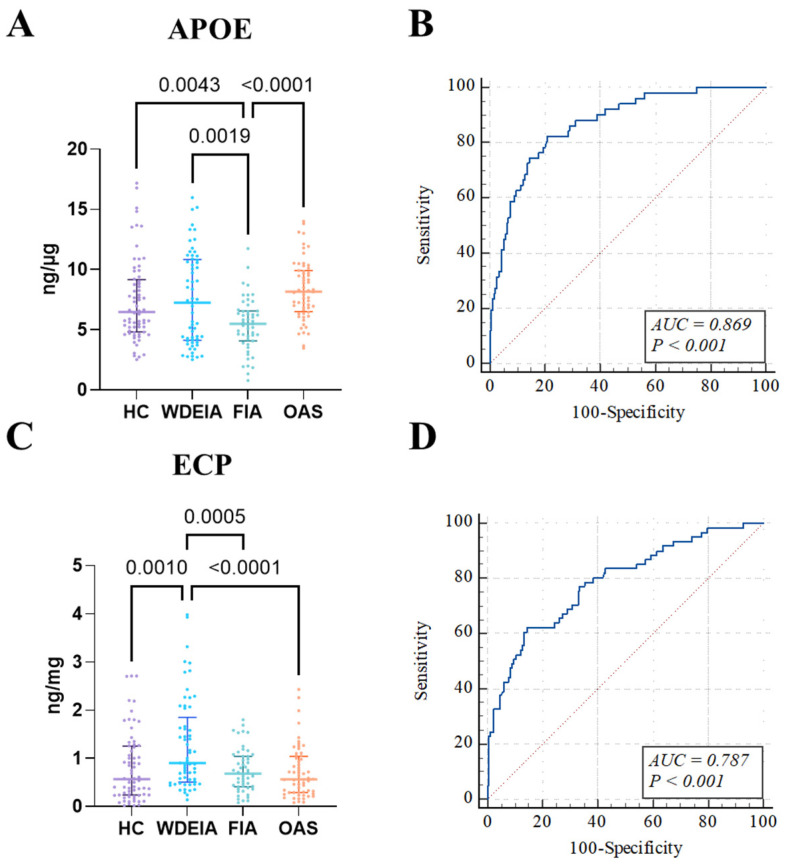
Validation of extracellular vesicles (EVs)-derived APOE and ECP levels. (**A**) EVs-derived apolipoprotein E (APOE) levels measured by ELISA in healthy controls (*n* = 68), WDEIA (*n* = 61), FIA (*n* = 51), and oral allergy syndrome (OAS) (*n* = 60) groups. (**B**) EVs-derived ECP levels measured by ELISA across different groups. (**C**) Receiver operating characteristic (ROC) curve evaluating the diagnostic performance of EV-derived APOE for distinguishing FIA patients from non-FIA (HC + OAS + WDEIA). (**D**) ROC curve evaluating the diagnostic performance of EV-derived ECP for distinguishing WDEIA patients from non-WDEIA (HC + OAS + FIA). “HC” refers to healthy control”; “WDEIA” refers to wheat-dependent exercise-induced anaphylaxis; “FIA” refers to food-induced anaphylaxis. Data are presented as mean ± SEM, one-way analysis of variance (ANOVA) followed by Tukey’s post hoc test to compare multiple groups.

To further evaluate the diagnostic potential of APOE and ECP in differentiating WDEIA from FIA, their levels were measured in serum-derived EVs using ELISA. The results showed that APOE was significantly decreased only in FIA (Figure 5A), while ECP was significantly elevated only in WDEIA (Figure 5B). ROC curve analysis demonstrated that APOE and ECP have moderate diagnostic value for distinguishing FIA from non-FIA conditions (including WDEIA, OAS, and healthy controls), and for distinguishing WDEIA from non-WDEIA conditions (including FIA, OAS, and healthy controls) (Figure 5C,D). The AUCs were 0.869 (95% CI: 0.820–0.909; cutoff: 5.37 ng/μg; sensitivity 82.35%, specificity 78.84%) and 0.787 (95% CI: 0.729–0.837; cutoff: 10.57 ng/μg; sensitivity 62.30%, specificity 85.47%), respectively.

## 3. Discussion

In this study, we performed a comprehensive proteomic analysis of serum-derived EVs to explore the underlying molecular differences between WDEIA and FIA. All samples were collected during the remission, at least one month after the most recent anaphylactic episode, ensuring that the observed proteomic profiles represent basal immune and metabolic states rather than acute responses. Using ultracentrifugation followed by mass spectrometry, we isolated and characterized EVs from patients and healthy controls, revealing both shared and distinct DEPs across the groups. Correlation analyses showed no significant associations between key EV-derived biomarkers and age, total/specific IgE levels, or sex, suggesting that the identified EV alterations are unlikely to be driven by these baseline demographic or sensitization factors. Collectively, these basal alterations may reflect immune dysregulation that predisposes individuals to systemic reactions upon allergen exposure, providing novel insights into EV-associated molecular changes and candidate biomarkers with potential diagnostic and predictive utility.

First, we validated the purity and quality of serum-derived EVs using SDS-PAGE, Western blotting for classical EV markers (ALIX and CD63), and NTA. Quantitative analyses revealed that WDEIA and FIA patients exhibited increased EV size and concentration compared with healthy controls, suggesting enhanced vesicle biogenesis or release [21]. These findings are consistent with prior studies demonstrating that cellular stress and immune activation can promote EV secretion [22]. Despite sampling during remission, increased EV production persisted, consistent with the relative stability of EVs [23] and the concept of sustained immune activation in food allergy as a chronic inflammatory condition [24]. These data indicate that altered EV profiles likely reflect persistent baseline immune dysregulation rather than transient changes from acute reactions.

TMT-based proteomics identified 583 EV-associated proteins, of which 83.2% overlapped with ExoCarta entries, confirming their vesicular origin. GO enrichment revealed proteins involved in immune responses, extracellular matrix components, and regulation of body fluid levels, relevant to leukocyte infiltration, vascular permeability, and hypotension in anaphylaxis [25,26,27]. Importantly, hierarchical clustering distinguished WDEIA, FIA, and healthy controls, though WDEIA and FIA exhibited overlapping DEPs, indicating shared inflammatory pathways. KEGG pathway analysis further implicated the complement and coagulation cascades, which are known to be central mediators of anaphylactic responses [28]. Interestingly, pathways related to regulation of cytoskeleton were also enriched, suggesting that EV-associated proteins may influence cellular structural dynamics [25], which may influence vascular permeability, immune cell migration, and tissue responses in the stable state, potentially increasing susceptibility to anaphylaxis.

A key finding was the significant reduction in EV-associated C1-INH in both WDEIA and FIA patients. C1-INH is a central regulator of the classical and lectin complement pathways by inhibiting C1r, C1s, and MASPs [29], and its dysfunction is well known to contribute to bradykinin-mediated vascular leakage in other disease contexts [30]. In our dataset, multiple complement-related proteins were increased, while C1-INH was reduced, suggesting dysregulated packaging into EVs during the basal state, potentially predisposing patients to systemic reactions. EV-derived C1-INH was higher in OAS patients than in WDEIA or FIA, correlating with clinical phenotype severity. ROC analyses indicated that EV-associated C1-INH could discriminate against anaphylaxis from non-anaphylactic conditions, highlighting its potential as a group-level biomarker.

Comparative analysis identified 76 non-overlapping DEPs between WDEIA and FIA, enriched in humoral immune responses and leukocyte-mediated immunity. PPI network analysis highlighted hub proteins, notably APOE and ECP. APOE is a multifunctional apolipoprotein traditionally known for its role in lipid metabolism and cardiovascular disease [31,32], and also modulates innate and adaptive immunity [33,34,35]. In our study, EV-associated APOE was specifically downregulated in FIA patients, consistent with prior reports in murine and human anaphylaxis models [36]. This may reflect altered capacity to respond to allergen-induced APOE depletion, indicating mechanistic differences between FIA and WDEIA, where cofactor-dependent mechanisms trigger systemic responses [37].

ECP (encoded by *RNASE3*), a major granule protein released by activated eosinophils, is widely recognized as a biomarker of eosinophil-associated inflammation and is commonly quantified in various biological fluids [38]. Elevated levels of ECP have been reported in T helper 2 (Th2)-driven (atopic) diseases, including a range of allergic conditions [39]. In our study, ECP levels within serum-derived EVs were significantly elevated at the basal level in patients with WDEIA, highlighting a distinctive baseline eosinophil-associated immune profile prior to allergen exposure. This elevated EV-associated ECP may reflect a basal eosinophilic inflammatory tendency, which could predispose individuals to exaggerated systemic reactions upon wheat ingestion, particularly in the presence of cofactors such as exercise. Exercise and other cofactors may facilitate systemic circulation of these EVs, thereby modulating the immune milieu and enhancing susceptibility to anaphylactic reactions.

A notable limitation of the discovery proteomic stage was the use of pooled serum samples for EV protein profiling. Sample pooling effectively minimized technical variability and facilitated robust exploratory detection of group level differences, which is a common and acceptable strategy in initial omics studies. However, pooling inherently prevents the evaluation of inter individual variability within each group and cannot account for potential outlier effects or heterogeneous protein expression patterns among individual patients. To overcome this limitation and strengthen the translational validity of our findings, we subsequently performed independent ELISA validation using fully individual serum samples in an expanded cohort. This individual level validation confirmed that the key biomarkers (C1 INH, APOE, and ECP) exhibited consistent expression trends observed in pooled proteomics, restored inter individual variation for statistical analysis, excluded outlier driven bias, and verified the diagnostic performance at the individual level.

Nevertheless, several additional limitations of this study should be acknowledged. First, the relatively small sample size—comprising predominantly adult patients—limits the generalizability of our results. The observed EV alterations are more likely to reflect disease-related immune dysregulation rather than individual-level diagnostic performance; thus, validation in larger, independent, and multicenter cohorts is required. Second, this study focused on comparisons between WDEIA and classical FIA and did not include other forms of anaphylaxis, such as drug-induced anaphylaxis, which may restrict the broader applicability of our conclusions. Third, clinical heterogeneity, the lack of standardized isolation methods, limited serum availability, and the use of baseline samples collected outside the acute phase precluded detailed stratified analyses, including correlations with reaction severity. Finally, the functional relevance of EV-associated APOE and ECP in anaphylaxis remains unclear, and prior studies have suggested that APOE, ECP, and C1-INH detected in EV preparations may represent serum-derived contaminants [40,41,42]. Further studies incorporating rigorous EV validation and mechanistic approaches are required to clarify their origin and biological roles.

## 4. Materials and Methods

### 4.1. Study Subjects and Sample Collection

A total of 240 participants were enrolled in this study (detailed clinical data are provided in Tables S1 and S2), including patients with WDEIA, FIA, oral allergy syndrome (OAS), and healthy controls. All diagnoses were made by an experienced allergist at the Department of Allergy, Peking Union Medical College Hospital (PUMCH, Beijing, China), and independently reviewed by another allergist, in accordance with established clinical guidelines [43,44]. All anaphylaxis patients had a well-documented clinical history of anaphylaxis together with positive skin prick testing and/or food-specific IgE to the implicated allergens. In the Chinese population, apart from wheat, fruits and vegetables represent the most common triggers of food allergy and food-induced anaphylaxis; accordingly, patients in both the FIA and OAS groups predominantly had fruit- or vegetable-related triggers, with most cases occurring in the context of pollen–food allergy syndrome. Written informed consent was obtained from all participants, and the study was approved by the institutional ethics committee of Peking Union Medical College Hospital (S-k976). For each patient, a 4 mL peripheral venous blood sample was collected in the morning under fasting conditions at least one month after their most recent allergic episode to minimize acute-phase confounding. Blood was drawn into serum-separating clot-activator tubes using a 21-gauge straight needle and allowed to clot at 18–25 °C for 15 min. Samples were then centrifuged at 4000 *g* for 10 min to obtain serum (ST 16, Thermo Fisher, Waltham, MA, USA). The serum supernatant was carefully inspected to exclude visible hemolysis or lipidemia, aliquoted into 1.5 mL tubes, and stored at −80 °C until further analysis to minimize freeze–thaw cycles.

In the discovery phase, serum samples were collected from 24 patients with WDEIA, 24 with FIA, and 56 healthy controls. For proteomic analysis, equal volumes of serum from individuals within each group were pooled to generate representative samples. In the validation phase, these same samples were included along with additional participants, expanding the cohort to 61 WDEIA patients, 51 FIA patients, and 68 healthy controls for individual-level analyses. These individual samples were used for ELISA-based quantification of candidate EV-associated proteins identified in the discovery phase. To further evaluate the diagnostic value and specificity of key biomarkers in distinguishing systemic anaphylaxis from localized allergic responses, an additional cohort of 60 patients with OAS was recruited during this phase.

### 4.2. EVs Isolation from Serum

For proteomics analysis, serum-derived EVs were isolated from pooled serum using ultracentrifugation, as previously described [19]. The pooled serum was prepared by combining 0.6 mL of aliquots from individual samples. A total volume of 12.5 mL of this pooled serum was utilized for each extraction via ultracentrifugation. Briefly, pooled serum from each group was first centrifuged at 10,000× *g* for 30 min at 4 °C using a Beckman Optima L-100XP 107 ultracentrifuge (Beckman Coulter, Brea, CA, USA) to remove debris. The supernatant was then transferred to ultracentrifuge tubes and subjected to ultracentrifugation at 110,000× *g* for 90 min at 4 °C. The resulting pellet was washed with 1 mL of PBS and resuspended in 40 μL of 8 M urea. The total protein concentration was determined using a NanoDrop spectrophotometer (Thermo Scientific, Waltham, MA, USA).

For validation of candidate proteins, EVs were isolated from individual sample using Total Exosome Isolation Reagent (from serum, Invitrogen, Carlsbad, CA, USA, 4478360), following the manufacturer’s protocol. In brief, a 300 μL aliquot of each serum sample was diluted 1:1 with an equal volume of PBS, followed by the addition of 0.2 volumes of the isolation reagent. The mixture was vortexed and incubated at 4 °C for 30 min, then centrifuged at 10,000× *g* for 30 min at room temperature. The resulting EV pellet was resuspended in PBS. Protein concentration of the isolated EVs was quantified using a BCA Protein Assay Kit (Thermo Scientific, Waltham, MA, USA, 23227) after lysis with RIPA buffer and stored at −80 °C until further analysis.

### 4.3. TMT Labeling

EVs-derived proteins were reduced with 10 mM DTT at 37 °C for 1 h and subsequently alkylated with 25 mM iodoacetamide for 1 h at room temperature in the dark. Proteins were then digested with a Trypsin/Lys-C mixture at a 1:25 (enzyme: substrate, w/w) ratio for 14 h at 37 °C. After digestion, samples were incubated in a 60 °C water bath for 30 min, followed by acidification with 1% trifluoroacetic acid (TFA) to adjust the pH to 1–2. The mixture was centrifuged at 12,000× *g* for 10 min to remove precipitates. Peptides were desalted using Oasis HLB reversed-phase cartridges (Waters Corporation, Milford, CT, USA), vacuum-dried for 1 h, and reconstituted in 50 μL of 200 mM triethylammonium bicarbonate buffer. TMT labeling was performed using the TMT Mass Tagging Kit (Thermo Scientific, Waltham, MA, USA) according to the manufacturer’s instructions. Briefly, 0.48 mg of TMT reagent dissolved in 25 μL of 99.9% acetonitrile was added to each peptide sample and incubated at room temperature for 1 h. The labeling reaction was quenched with 8 μL of 5% hydroxylamine for 15 min. Peptides from WDEIA, FIA, and healthy control groups were labeled with TMT-129, TMT-131, and TMT-126, respectively. The labeled peptides were pooled, desalted, vacuum-dried, and reconstituted in 200 μL of 0.1% TFA for subsequent analysis.

### 4.4. LC-MS/MS

The labeled peptide mixtures were separated using high performance liquid chromatography prior to LC-MS/MS analysis. Fractionation was performed on a Waters X bridge BEH300 C18 column (4.6 × 250 mm, 2.5 μm) using a linear gradient with a flow rate of 1.0 mL/min. LC-MS/MS was conducted on a Thermo Q Exactive Benchtop mass spectrometer (Thermo Scientific, Waltham, MA, USA) coupled with an UltiMate 3000 RSLCnano System (Thermo Scientific, Waltham, MA, USA). Peptide separation was performed on an in-house packed analytical column (75 μm × 15 cm, C18, 2 μm particle size) using a 120 min linear gradient at a flow rate of 300 nL/min. The mass spectrometer was operated in data-dependent acquisition mode using Xcalibur 4.1 software. A single full MS scans were acquired in the Orbitrap (*m*/*z* 350–1550) with a resolution of 120,000 at *m*/*z*. The most intense precursor ions were selected for higher-energy collisional dissociation (HCD) fragmentation with a normalized collision energy of 35%.

### 4.5. Nanoparticle Tracking Analysis (NTA)

The EVs isolated from pooled serum samples were diluted 20,000-fold to achieve optimal measurement conditions. A total volume of approximately 300 μL of the diluted EV suspension was introduced into the sample chamber of a Nanosight LM10 system (Malvern Panalytical, Amesbury, UK). The system utilized a 635 nm laser to illuminate the particles, enabling the visualization and recording of their Brownian motion for a duration of 60 s. Each sample was measured in triplicate. The final data were analyzed with NTA 3.2 software (Nanosight, Amesbury, Wiltshire, UK).

### 4.6. Western Blot Analysis

Serum EVs were applied for immunoblotting. For Western blot analysis, the lysed EVs (15 μg total protein) were separated by SDS-PAGE. Proteins were transferred onto a polyvinylidene difluoride (PVDF) membrane using wet transfer apparatus. The membrane was blocked with 5% non-fat milk in TBST (Tris-buffered saline with 0.05% Tween-20) for 1 h at room temperature. The membrane was incubated overnight at 4 °C with a primary antibody (CD63, Cell Signaling Technology Inc., Danvers, MA, USA, 52090; ALIX, Cell Signaling Technology Inc., Danvers, MA, USA, 92880). After washing the membrane with TBST, it was incubated with a horseradish peroxidase (HRP)-conjugated Goat Anti-Rabbit antibody (Jackson ImmunoResearch Laboratories Inc., West Grove, PA, USA, 111-035-003) for 1 h at room temperature. The protein bands were visualized using ECL reagents.

### 4.7. Bioinformatics Analysis

Raw LC-MS/MS data were processed using Proteome Discoverer software suite (version 2.2, Thermo Fisher, Waltham, MA, USA) with the SEQUEST search engine. Spectra were searched against the UniProt/SwissProt human proteome database. Relative quantification of proteins was performed based on TMT reporter ion intensities extracted by Proteome Discoverer. Reporter ion intensities were normalized across all channels to correct for sample loading differences. Proteins with fold change ≥ 1.5 or ≤0.67 were considered differentially expressed proteins (DEPs).

For functional annotation, Gene Ontology (GO) classification—including biological process, molecular function, and cellular component—was conducted using FunRich (version 3.0) and WebGestalt (http://www.webgestalt.org/, accessed on 21 October 2025). KEGG and WikiPathways enrichment analyses were also performed to identify relevant biological pathways. Enrichment results with *p* < 0.05 and at least four mapped proteins were considered significant. Protein–protein interaction (PPI) networks of DEPs were constructed using the STRING database (https://string-db.org, accessed on 21 October 2025). The resulting interaction networks were visualized and analyzed using Cytoscape software (version 3.6.1).

### 4.8. ELISA

EVs were isolated from individual serum samples using the Exosome Isolation Reagent. Levels of target proteins within EVs were quantified using commercial ELISA kits following the manufacturer’s protocols, including Human Plasma Protease C1 Inhibitor (SERPING1) ELISA Kit (CSB-EL021086HU, Cusabio, Wuhan, China), Human Apolipoprotein E (ApoE) ELISA Kit (CSB-E09748h, Cusabio, Wuhan, China), and Human Eosinophil Cationic Protein (ECP) ELISA Kit (CSB-E11729h-IS, Cusabio, Wuhan, China).

### 4.9. Statistical Analysis

All statistical analyses were performed using IBM SPSS Statistics software (version 23.0; IBM, Armonk, NY, USA) and GraphPad Prism (version 9.0; GraphPad Software, San Diego, CA, USA). Data are presented as mean ± standard error of the mean (SEM) or median as appropriate. Comparisons among multiple groups were conducted using the Kruskal–Wallis’s test for nonparametric data. For parametric analyses, we employed one-way analysis of variance (ANOVA) followed by Tukey’s post hoc test to compare multiple groups. Pearson correlation analysis was used to evaluate correlations between variables. Receiver operating characteristic (ROC) curve analysis was performed to assess the diagnostic performance of selected biomarkers. A two-tailed *p* value < 0.05 was considered statistically significant.

## 5. Conclusions

In summary, our study demonstrates that basal alterations in complement-related proteins within serum-derived EVs represent a shared molecular feature across different types of food-related anaphylaxis, while EV-associated C1-INH, APOE, and ECP display subtype-specific expression patterns that enable discrimination between WDEIA and classical FIA. These findings support EV profiling as a novel, minimally invasive approach for the diagnosis of food-related anaphylaxis subtypes in clinical practice and suggest that such EV-based signatures may ultimately assist in identifying high-risk individuals at baseline, thereby facilitating risk stratification and preventive management strategies prior to severe reactions. Moreover, the presence of disease-associated EV alterations at baseline suggests that EVs may participate in the underlying pathophysiology of anaphylaxis, raising the possibility that therapeutic strategies aimed at modulating EV composition or function could be explored in the future. Although such applications remain preliminary, our results provide a rationale for further investigation of EVs as both diagnostic biomarkers and potential targets for intervention in food-related anaphylaxis.

## Data Availability

The original contributions presented in the study are included in the article/Supplementary Materials. Further inquiries can be directed at the corresponding author.

## Supplementary Materials

The following supporting information can be downloaded at: https://www.mdpi.com/article/10.3390/ijms27114732/s1.

## Abbreviations

The following abbreviations are used in this manuscript:

FIA	Food-induced anaphylaxis
WDEIA	Wheat-dependent exercise-induced anaphylaxis
EV	Extracellular vesicle
OAS	Oral allergy syndrome
C1-INH	C1-inhibitor
APOE	Apolipoprotein E
ECP	Eosinophil cationic protein
IgE	Immunoglobulin E
TMT	Tandem mass tag
TFA	Trifluoroacetic acid
DEPs	Differentially expressed proteins
GO	Gene Ontology
ROC	Receiver operating characteristic
