# Supplementary material for: Baseline Immune Signatures in Serum Extracellular Vesicles Distinguish Food-Induced from Wheat-Dependent Exercise-Induced Anaphylaxis
Source: Int J Mol Sci. 2026 May 25;27(11):4732. doi: 10.3390/ijms27114732 (PMC13256944; doi:10.3390/ijms27114732)
Supplement: Supplementary file 1 [file ijms-27-04732-s001.zip › ijms-4251640-supplementary.pdf]

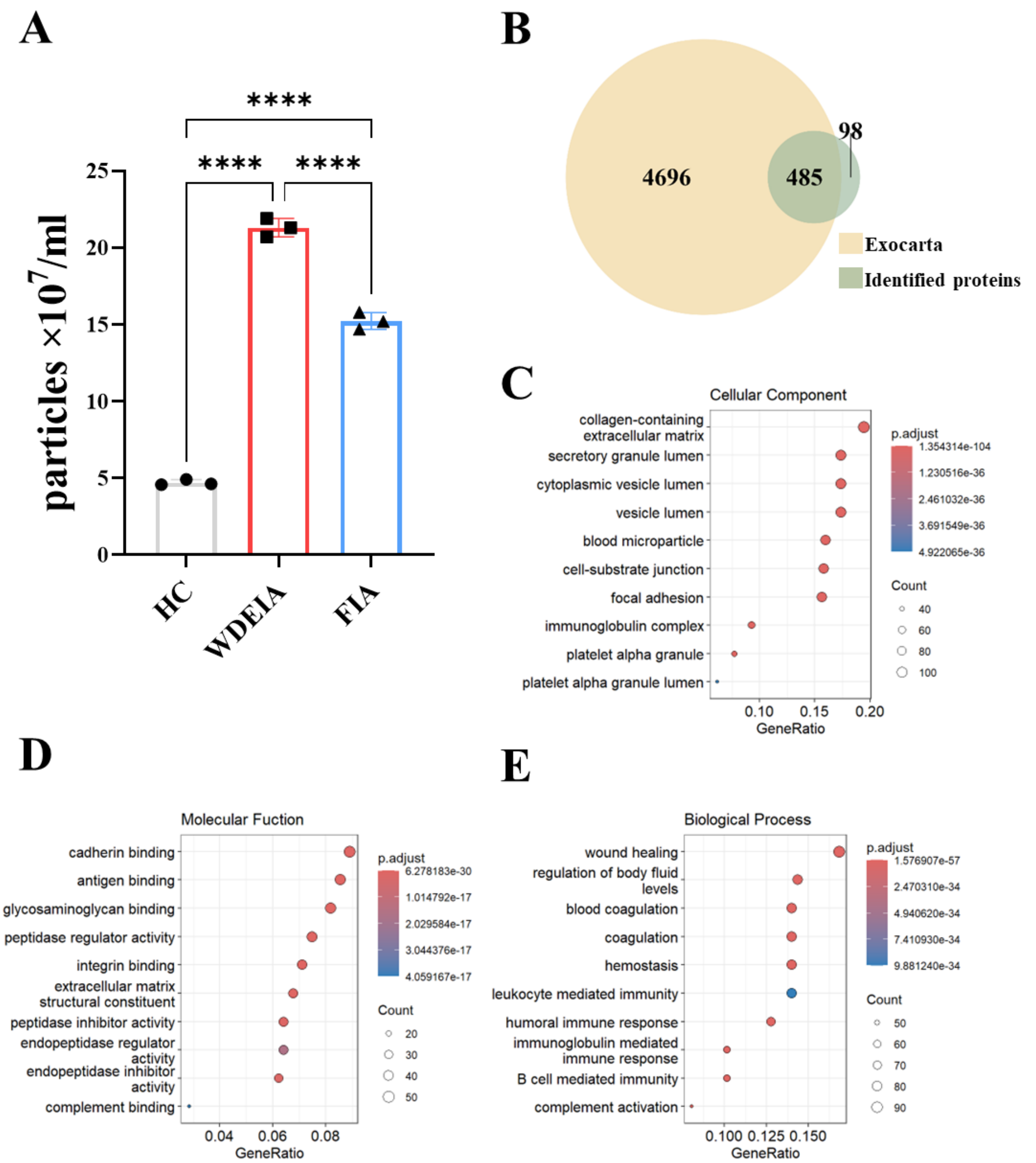

Figure S1. Characterization and functional enrichment of extracellular vesicle (EV) proteins from pooled serum. **(A)** Comparison of mean EVs particle concentration among healthy controls, WDEIA, and FIA groups (Each group: n =3, representing three independent measurements). **(B)** Venn diagram showing the overlap between identified EV proteins and those listed in the ExoCarta database. **(C-E)** Gene ontology (GO) analysis of EVs proteins classified by cellular component (C), molecular function(D), and biological process(E). "HC" refers to healthy control"; "WDEIA" refers to wheat-dependent exercise-induced anaphylaxis; "FIA" refers to food-induced anaphylaxis.

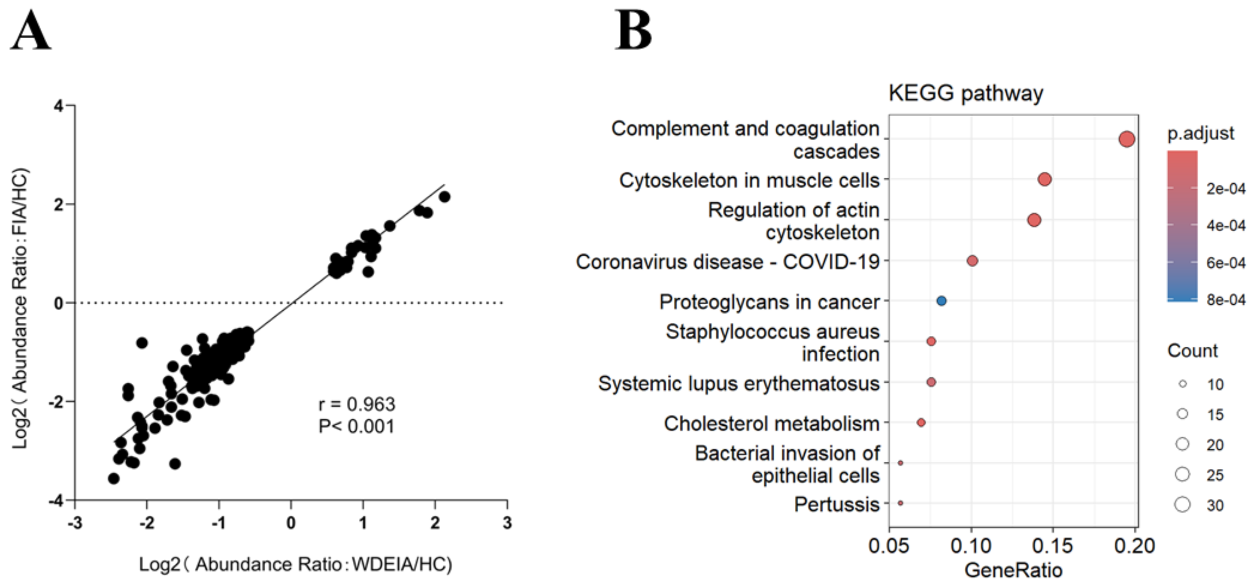

Figure S2. Correlation and pathway enrichment analysis of shared differentially expressed proteins (DEPs) in WDEIA and FIA. (A) Pearson correlation analysis of shared DEPs between WDEIA and FIA groups, showing high concordance in protein expression profiles. (B) KEGG pathway enrichment analysis of the shared DEPs, highlighting their involvement in complement and coagulation cascades pathway. “WDEIA” refers to wheat-dependent exercise-induced anaphylaxis; “FIA” refers to food-induced anaphylaxis.

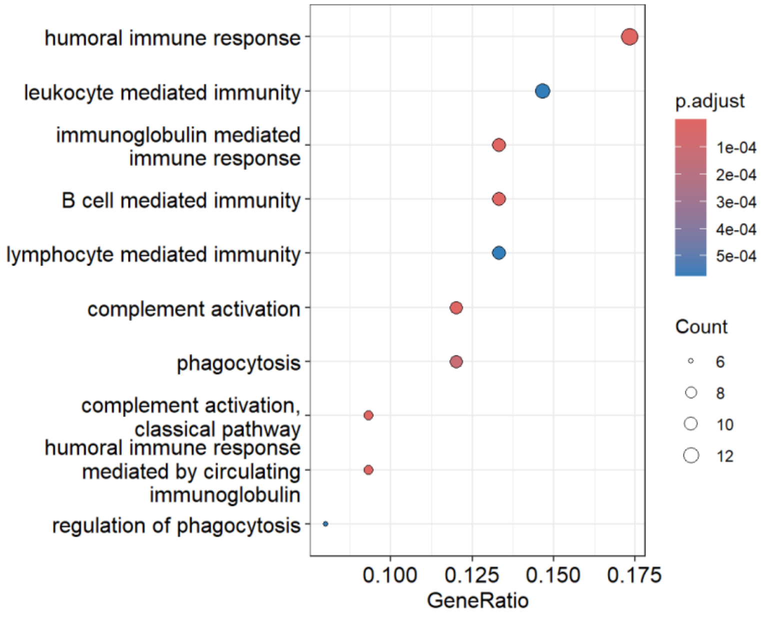

Figure S3. KEGG pathway enrichment analysis of non-overlapping differentially expressed proteins (DEPs) between WDEIA and FIA. “WDEIA” refers to wheat-dependent exercise-induced anaphylaxis; “FIA” refers to food-induced anaphylaxis.

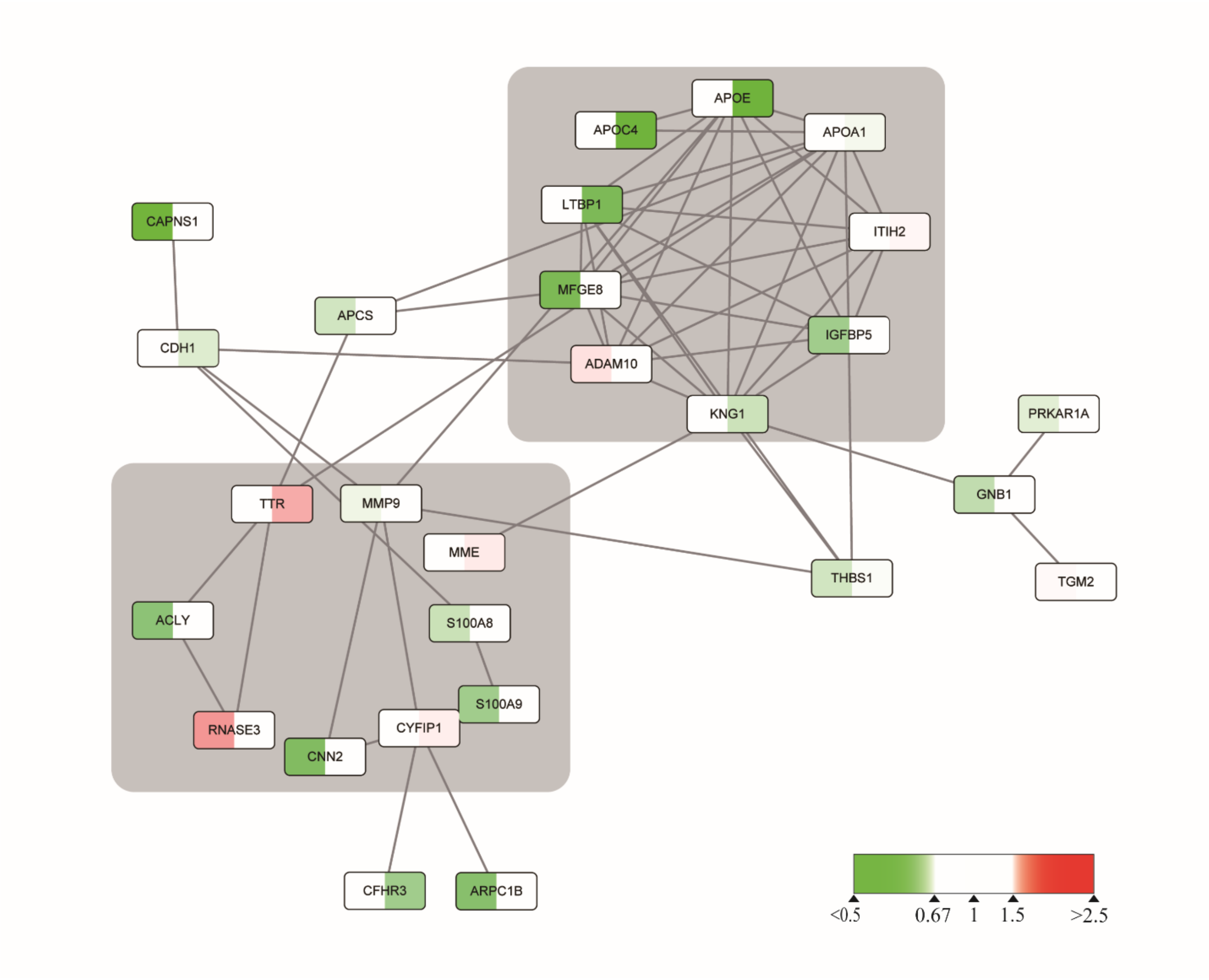

Figure S4. Protein–protein interaction (PPI) network of non-overlapping DEPs between WDEIA and FIA. For each identified protein, the comparison of WDEIA versus healthy controls is displayed on the left, and FIA versus healthy controls is shown on the right. Proteins with higher expression levels are indicated with a red background, whereas downregulated proteins are indicated with a green background. “WDEIA” refers to wheat-dependent exercise-induced anaphylaxis; “FIA” refers to food-induced anaphylaxis.

Table S1. Clinical and demographic characteristics of patients

	Healthy control (n = 68)	WDEIA (n = 61)	FIA (n = 51)	OAS (n = 60)	P value
Male, n (%)	39 (57%)	31 (51%)	22 (43%)	27 (45%)	0.387
Age, mean (range)	35.8 (12-67)	35.1(13-62)	31.9 (10-67)	32.2 (11-67)	0.237
Allergic Rhinitis, n (%)	-	15 (24.6%)	43 (84.3%)	53 (88.3%)	<0.001
Allergic Asthma, n (%)	-	3 (4.9%)	12 (23.5%)	17 (28.3%)	0.002
Chronic Urticaria, n (%)	-	49 (80.3%)	7 (13.7%)	8 (13.3%)	<0.001
Total IgE, KU/L, median, (range)	25.4 (2-66.6)	217 (48.8-1576)	221 (11.5-2537)	271 (18.2-1249)	<0.001

WDEIA: Wheat-dependent exercise-induced anaphylaxis; FIA: Food-induced anaphylaxis; OAS: Oral allergy syndrome.

Table S2. Detail information about patients recruited for the experiments

ID (W/F/O/C)	Age (years)	Gender (Male/Female)	Diagnosis	Allergic Rhinitis	Allergic Asthma	Chronic Urticaria	Total IgE KU/L	culprit	Cofactor	sIgE* kUA/L	SPT#	TMT	Pooled sample
W1	51	Male	WDEIA	N	N	Y	363	wheat	exercise	0.70	NT	N	N
W2	26	Female	WDEIA	N	N	N	196	wheat	exercise	3.63	NT	N	N
W3	48	Male	WDEIA	N	N	Y	51.3	wheat	exercise	1.30	NT	N	N
W4	22	Male	WDEIA	N	N	N	186	wheat	exercise	1.20	NT	N	N
W5	35	Male	WDEIA	Y	N	Y	139	wheat	exercise	2.08	NT	N	N
W6	46	Male	WDEIA	N	N	Y	215	wheat	exercise	4.17	NT	N	N
W7	43	Male	WDEIA	N	N	Y	138	wheat	exercise	5.83	NT	N	N
W8	27	Female	WDEIA	N	N	Y	1047	wheat	exercise	16.9	NT	N	N
W9	18	Male	WDEIA	Y	N	N	60.3	wheat	exercise	1.28	NT	N	N
W10	33	Female	WDEIA	N	N	Y	166	wheat	exercise	8.04	+	N	N
W11	20	Male	WDEIA	N	N	Y	326	wheat	exercise	10.7	+	N	N
W12	45	Male	WDEIA	N	N	Y	123	wheat	exercise	3.18	NT	N	N
W13	40	Male	WDEIA	N	N	Y	640	wheat	exercise	1.55	NT	Y	Y
W14	38	Male	WDEIA	Y	N	Y	98.1	wheat	exercise	0.72	NT	Y	Y
W15	13	Female	WDEIA	N	N	N	758	wheat	exercise	0.70	+	Y	Y
W16	41	Male	WDEIA	N	N	Y	60.3	wheat	exercise	0.95	NT	Y	Y
W17	47	Female	WDEIA	N	N	Y	148	wheat	exercise	1.68	NT	Y	Y
W18	13	Male	WDEIA	Y	N	N	74.3	wheat	exercise	3.63	NT	Y	Y
W19	14	Female	WDEIA	Y	Y	N	173	wheat	exercise	1.30	NT	Y	Y
W20	22	Female	WDEIA	N	N	Y	1017	wheat	exercise	8.04	+	Y	Y

ID (W/F/O/C)	Age (years)	Gender (Male/Female)	Diagnosis	Allergic Rhinitis	Allergic Asthma	Chronic Urticaria	Total IgE KU/L	culprit	Cofactor	sIgE* kUA/L	SPT#	TMT	Pooled sample
W21	34	Female	WDEIA	N	N	Y	114	wheat	exercise	4.09	NT	Y	Y
W22	23	Male	WDEIA	N	N	Y	105	wheat	alcohol	11.4	+	Y	Y
W23	17	Male	WDEIA	Y	N	N	291	wheat	exercise	11.7	NT	Y	Y
W24	24	Female	WDEIA	N	N	Y	328	wheat	exercise	1.55	NT	Y	Y
W25	35	Male	WDEIA	N	N	Y	1475	wheat	exercise	1.33	NT	Y	Y
W26	40	Female	WDEIA	N	N	Y	175	wheat	exercise	7.34	NT	Y	Y
W27	62	Male	WDEIA	Y	Y	Y	343	wheat	exercise	1.42	NT	Y	Y
W28	21	Female	WDEIA	N	N	N	328	wheat	exercise	2.33	NT	Y	Y
W29	45	Male	WDEIA	Y	N	Y	294	wheat	exercise	0.61	NT	Y	Y
W30	41	Female	WDEIA	N	N	N	55.7	wheat	exercise	2.55	NT	Y	Y
W31	59	Male	WDEIA	N	N	Y	82.5	wheat	exercise	6.11	NT	Y	Y
W32	26	Female	WDEIA	N	N	Y	203	wheat	exercise	1.68	NT	Y	Y
W33	36	Female	WDEIA	Y	N	Y	1169	wheat	exercise	5.32	NT	Y	Y
W34	55	Male	WDEIA	N	N	Y	1576	wheat	exercise	2.12	NT	Y	Y
W35	60	Male	WDEIA	N	N	Y	836	wheat	exercise	7.34	+	Y	Y
W36	48	Female	WDEIA	Y	N	Y	75	wheat	exercise	0.44	NT	Y	Y
W37	20	Male	WDEIA	N	N	N	97.3	wheat	exercise	0.95	NT	N	N
W38	24	Male	WDEIA	N	N	Y	74.9	wheat	exercise	2.19	NT	N	N
W39	51	Female	WDEIA	N	N	Y	178	wheat	exercise	0.83	NT	N	N
W40	34	Male	WDEIA	N	N	Y	48.8	wheat	exercise	2.68	+	N	N
W41	26	Female	WDEIA	Y	Y	Y	52.5	wheat	exercise	4.09	NT	N	N

ID (W/F/O/C)	Age (years)	Gender (Male/Female)	Diagnosis	Allergic Rhinitis	Allergic Asthma	Chronic Urticaria	Total IgE KU/L	culprit	Cofactor	sIgE* kUA/L	SPT#	TMT	Pooled sample
W42	42	Male	WDEIA	Y	N	Y	497	wheat	exercise	2.33	NT	N	N
W43	35	Male	WDEIA	N	N	Y	595	wheat	exercise	2.17	NT	N	N
W44	26	Female	WDEIA	N	N	Y	422	wheat	exercise	4.28	NT	N	N
W45	30	Male	WDEIA	N	N	Y	968	wheat	exercise	3.46	NT	N	N
W46	16	Male	WDEIA	Y	N	N	514	wheat	exercise	1.12	NT	N	N
W47	46	Male	WDEIA	N	N	Y	365	wheat	exercise	7.35	NT	N	N
W48	40	Male	WDEIA	N	N	Y	214	wheat	exercise	5.52	NT	N	N
W49	35	Female	WDEIA	Y	N	Y	227	wheat	exercise	1.58	NT	N	N
W50	22	Female	WDEIA	N	N	Y	1035	wheat	exercise	0.72	+	N	N
W51	34	Female	WDEIA	N	N	Y	124	wheat	exercise	3.53	NT	N	N
W52	31	Female	WDEIA	N	N	Y	146	wheat	exercise	1.93	NT	N	N
W53	32	Female	WDEIA	N	N	Y	1370	wheat	exercise	12.2	NT	N	N
W54	23	Female	WDEIA	N	N	N	1077	wheat	exercise	1.18	NT	N	N
W55	47	Female	WDEIA	N	N	Y	217	wheat	alcohol	2.18	NT	N	N
W56	57	Female	WDEIA	N	N	Y	615	wheat	exercise	5.69	NT	N	N
W57	40	Female	WDEIA	N	N	Y	394	wheat	exercise	2.54	NT	N	N
W58	33	Female	WDEIA	N	N	Y	896	wheat	exercise	5.14	NT	N	N
W59	40	Female	WDEIA	N	N	Y	52.4	wheat	exercise	0.77	NT	N	N
W60	48	Male	WDEIA	Y	N	Y	338	wheat	exercise	1.92	NT	N	N
W61	43	Female	WDEIA	N	N	Y	460	wheat	exercise	1.42	NT	N	N
F1	36	Male	FIA	Y	N	N	240	apple	-	1.21	NT	Y	Y
F2	32	Female	FIA	Y	N	N	334	peach	-	2.38	+	Y	Y

ID (W/F/O/C)	Age (years)	Gender (Male/Female)	Diagnosis	Allergic Rhinitis	Allergic Asthma	Chronic Urticaria	Total IgE KU/L	culprit	Cofactor	sIgE* kUA/L	SPT#	TMT	Pooled sample
F3	23	Female	FIA	Y	N	N	594	peach	-	11.7	NT	Y	Y
F4	17	Male	FIA	Y	Y	N	653	peach	-	13.7	NT	Y	Y
F5	26	Female	FIA	Y	N	N	525	mango	-	14.8	NT	Y	Y
F6	19	Female	FIA	Y	N	N	103	peach	-	5.81	NT	Y	Y
F7	18	Female	FIA	Y	N	N	276	peach	-	33.2	NT	Y	Y
F8	23	Male	FIA	Y	Y	N	2537	cheery	-	3.28	NT	Y	Y
F9	10	Male	FIA	N	N	N	24.6	celery	-	4.58	NT	Y	Y
F10	35	Male	FIA	Y	Y	Y	1485	mango	-	2.38	+	Y	Y
F11	47	Male	FIA	Y	N	N	216	peach	-	28.5	NT	Y	Y
F12	13	Female	FIA	Y	Y	N	816	peach	-	1.29	NT	Y	Y
F13	30	Male	FIA	Y	Y	N	494	peach	-	31.6	+	Y	Y
F14	62	Female	FIA	Y	Y	Y	1257	peach	-	13.2	NT	Y	Y
F15	49	Female	FIA	Y	N	N	184	hazel	-	9.73	NT	Y	Y
F16	12	Female	FIA	N	N	N	11.5	walnut	-	14.1	NT	Y	Y
F17	46	Female	FIA	Y	N	Y	439	apple	-	22.5	+	Y	Y
F18	17	Male	FIA	Y	N	N	396	peanut	-	3.34	NT	Y	Y
F19	48	Male	FIA	Y	N	Y	116	legume	-	21.5	NT	Y	Y
F20	16	Male	FIA	Y	N	N	192	peach	-	31.6	NT	Y	Y
F21	11	Female	FIA	N	N	N	82.2	soybea	-	0.97	NT	N	N
F22	19	Female	FIA	Y	N	N	186	peach	-	42.3	NT	N	N
F23	57	Female	FIA	Y	Y	N	810	celery	-	2.84	NT	N	N

ID (W/F/O/C)	Age (years)	Gender (Male/Female)	Diagnosis	Allergic Rhinitis	Allergic Asthma	Chronic Urticaria	Total IgE KU/L	culprit	Cofactor	sIgE* kUA/L	SPT#	TMT	Pooled sample
F24	44	Female	FIA	Y	Y	N	616	cheery	-	1.74	NT	N	N
F25	36	Female	FIA	Y	Y	N	517	peach	-	13.8	NT	N	N
F26	56	Male	FIA	N	N	N	63.6	fish	-	20.8	NT	N	N
F27	59	Female	FIA	Y	N	N	498	celery	-	3.47	NT	N	N
F28	26	Female	FIA	N	N	N	58.6	peach	-	8.92	NT	N	N
F29	20	Male	FIA	Y	N	N	357	peach	-	12.5	NT	Y	Y
F30	14	Male	FIA	Y	N	N	153	peach	-	20.1	NT	Y	Y
F31	25	Male	FIA	Y	N	N	552	lentils	-	4.06	NT	Y	Y
F32	26	Female	FIA	Y	N	N	243	broccol	-	1.28	NT	Y	Y
F33	27	Male	FIA	Y	N	N	210	peach	-	9.74	NT	N	N
F34	51	Male	FIA	Y	N	Y	194	peach	-	5.67	NT	N	N
F35	67	Female	FIA	Y	Y	N	494	apple	-	16.7	NT	N	N
F36	46	Male	FIA	Y	N	N	189	peach	-	7.24	NT	N	N
F37	29	Male	FIA	Y	N	N	216	celery	-	8.80	NT	N	N
F38	51	Male	FIA	Y	N	Y	104	apple	-	2.05	NT	N	N
F39	29	Female	FIA	Y	N	N	1257	peach	-	24.2	NT	N	N
F40	19	Male	FIA	Y	N	N	191	peach	-	3.91	NT	N	N
F41	12	Female	FIA	Y	N	N	221	peach	-	4.00	NT	N	N
F42	21	Female	FIA	Y	Y	N	769	peach	-	15.7	NT	N	N
F43	25	Female	FIA	N	N	N	38.7	peach	-	8.14	NT	N	N
F44	34	Female	FIA	Y	N	Y	254	cherry	-	19.4	NT	N	N
F45	39	Female	FIA	N	N	N	93.9	peach	-	1.92	NT	N	N

ID (W/F/O/C)	Age (years)	Gender (Male/Female)	Diagnosis	Allergic Rhinitis	Allergic Asthma	Chronic Urticaria	Total IgE KU/L	culprit	Cofactor	sIgE* kUA/L	SPT#	TMT	Pooled sample
F46	53	Female	FIA	Y	N	N	168	peach	-	9.61	NT	N	N
F47	22	Female	FIA	Y	N	N	132	hazel	-	14.2	NT	N	N
F48	45	Male	FIA	N	N	N	43.3	peach	-	5.08	NT	N	N
F49	35	Female	FIA	Y	N	N	189	peach	-	3.34	NT	N	N
F50	29	Female	FIA	Y	N	N	99.4	peach	-	7.83	NT	N	N
F51	21	Male	FIA	Y	Y	N	439	peach	-	23.7	NT	N	N
O1	30	Female	OAS	Y	N	N	447	orange	-	0.46	NT	N	N
O2	28	Male	OAS	Y	N	N	120	peach	-	4.11	NT	N	N
O3	18	Male	OAS	Y	Y	N	660	peach	-	7.20	NT	N	N
O4	20	Male	OAS	Y	N	N	435	peach	-	2.75	NT	N	N
O5	14	Male	OAS	Y	N	N	67	peach	-	3.51	NT	N	N
O6	36	Female	OAS	Y	N	N	334	peach	-	31.8	NT	N	N
O7	40	Female	OAS	Y	N	Y	372	peach	-	5.47	NT	N	N
O8	14	Male	OAS	Y	Y	N	1249	peach	-	2.38	NT	N	N
O9	37	Male	OAS	Y	N	N	232	peach	-	7.06	NT	N	N
O10	52	Female	OAS	Y	Y	Y	643	peach	-	12.7	NT	N	N
O11	23	Female	OAS	Y	N	N	587	peach	-	13.0	+	N	N
O12	67	Female	OAS	Y	Y	N	683	peach	-	2.37	NT	N	N
O13	37	Female	OAS	Y	N	N	574	peach	-	4.21	NT	N	N
O14	49	Female	OAS	Y	N	N	239	apple	-	10.5	NT	N	N
O15	41	Female	OAS	Y	N	N	429	peach	-	7.83	NT	N	N

ID (W/F/O/C)	Age (years)	Gender (Male/Female)	Diagnosis	Allergic Rhinitis	Allergic Asthma	Chronic Urticaria	Total IgE KU/L	culprit	Cofactor	sIgE* kUA/L	SPT#	TMT	Pooled sample
O16	51	Female	OAS	Y	N	N	217	peach	-	7.92	NT	N	N
O17	39	Female	OAS	Y	N	N	384	peach	-	15.0	+	N	N
O18	61	Female	OAS	Y	Y	N	731	peach	-	NT	+	N	N
O19	34	Male	OAS	Y	Y	N	484	apple	-	4.54	NT	N	N
O20	61	Male	OAS	Y	N	N	216	peach	-	9.18	NT	N	N
O21	21	Female	OAS	Y	Y	N	707	peach	-	6.77	NT	N	N
O22	21	Female	OAS	Y	N	N	306	peach	-	10.2	NT	N	N
O23	27	Female	OAS	Y	Y	N	521	peach	-	2.35	NT	N	N
O24	32	Male	OAS	Y	N	N	142	peach	-	1.67	+	N	N
O25	23	Female	OAS	Y	Y	N	387	peach	-	3.78	NT	N	N
O26	34	Female	OAS	Y	N	N	256	peach	-	14.6	NT	N	N
O27	42	Female	OAS	Y	N	Y	241	apple	-	NT	+	N	N
O28	43	Male	OAS	Y	N	N	114	peach	-	4.21	NT	N	N
O29	21	Male	OAS	Y	N	N	261	peach	-	7.06	NT	N	N
O30	23	Female	OAS	N	N	N	38.4	peach	-	5.08	+	N	N
O31	27	Female	OAS	Y	Y	N	652	peach	-	7.83	NT	N	N
O32	26	Male	OAS	N	N	N	32.2	peach	-	7.06	NT	N	N
O33	14	Female	OAS	Y	Y	N	1202	peach	-	20.7	NT	N	N
O34	30	Female	OAS	Y	N	N	52.1	apple	-	NT	+	N	N
O35	23	Female	OAS	Y	N	N	208	peach	-	18.7	NT	N	N
O36	36	Male	OAS	Y	N	Y	134	peach	-	9.16	NT	N	N
O37	15	Male	OAS	Y	Y	N	949	peach	-	14.8	NT	N	N

ID (W/F/O/C)	Age (years)	Gender (Male/Female)	Diagnosis	Allergic Rhinitis	Allergic Asthma	Chronic Urticaria	Total IgE KU/L	culprit	Cofactor	sIgE* kUA/L	SPT#	TMT	Pooled sample
O38	28	Female	OAS	N	N	N	36.9	apple	-	3.05	NT	N	N
O39	31	Male	OAS	Y	Y	N	597	peach	-	7.89	+	N	N
O40	11	Female	OAS	Y	N	N	342	peach	-	11.6	NT	N	N
O41	31	Female	OAS	Y	Y	N	584	peach	-	4.27	NT	N	N
O42	34	Female	OAS	N	N	N	53.2	peach	-	2.91	NT	N	N
O43	40	Female	OAS	Y	Y	N	287	peach	-	NT	+	N	N
O44	59	Female	OAS	Y	N	Y	65.1	peach	-	5.08	NT	N	N
O45	16	Male	OAS	Y	N	N	99.9	peach	-	6.31	NT	N	N
O46	25	Male	OAS	Y	N	N	137	peach	-	3.88	+	N	N
O47	39	Female	OAS	N	N	N	36.9	apple	-	4.69	NT	N	N
O48	33	Female	OAS	N	N	N	18.2	peach	-	2.17	NT	N	N
O49	26	Male	OAS	Y	N	N	284	peach	-	20.8	NT	N	N
O50	27	Male	OAS	Y	N	N	97.9	peach	-	NT	+	N	N
O51	58	Female	OAS	Y	N	Y	114	peach	-	8.35	NT	N	N
O52	32	Female	OAS	Y	N	Y	39.4	peach	-	6.09	NT	N	N
O53	18	Male	OAS	Y	N	N	281	peach	-	24.7	+	N	N
O54	16	Male	OAS	Y	N	N	317	peach	-	21.6	NT	N	N
O55	45	Male	OAS	Y	Y	N	490	apple	-	9.08	NT	N	N
O56	31	Male	OAS	Y	N	N	186	peach	-	12.6	NT	N	N
O57	32	Male	OAS	N	N	N	33.5	peach	-	7.57	NT	N	N
O58	33	Male	OAS	Y	N	N	81.1	peach	-	4.93	NT	N	N

ID (W/F/O/C)	Age (years)	Gender (Male/Female)	Diagnosis	Allergic Rhinitis	Allergic Asthma	Chronic Urticaria	Total IgE KU/L	culprit	Cofactor	sIgE* kUA/L	SPT#	TMT	Pooled sample
O59	29	Male	OAS	Y	Y	N	557	apple	-	14.6	NT	N	N
O60	27	Male	OAS	Y	N	Y	162	peach	-	6.83	NT	N	N
C1	29	Male	Healthy	N	N	N	26.3	N	-	NT	NT	Y	Y
C2	26	Female	Healthy	N	N	N	66.6	N	-	NT	NT	Y	Y
C3	28	Female	Healthy	N	N	N	59.5	N	-	NT	NT	Y	Y
C4	44	Female	Healthy	N	N	N	12.1	N	-	NT	NT	Y	Y
C5	12	Female	Healthy	N	N	N	28.4	N	-	NT	NT	Y	Y
C6	35	Male	Healthy	N	N	N	36.5	N	-	NT	NT	Y	Y
C7	14	Male	Healthy	N	N	N	19.5	N	-	NT	NT	Y	Y
C8	34	Female	Healthy	N	N	N	18.4	N	-	NT	NT	Y	Y
C9	27	Female	Healthy	N	N	N	38.7	N	-	NT	NT	Y	Y
C10	32	Male	Healthy	N	N	N	15.9	N	-	NT	NT	Y	Y
C11	18	Male	Healthy	N	N	N	29.3	N	-	NT	NT	Y	Y
C12	26	Male	Healthy	N	N	N	38.3	N	-	NT	NT	Y	Y
C13	25	Male	Healthy	N	N	N	52.3	N	-	NT	NT	Y	Y
C14	60	Male	Healthy	N	N	N	19.6	N	-	NT	NT	Y	Y
C15	33	Male	Healthy	N	N	N	44.8	N	-	NT	NT	Y	Y
C16	65	Male	Healthy	N	N	N	33.1	N	-	NT	NT	Y	Y
C17	31	Female	Healthy	N	N	N	57.2	N	-	NT	NT	Y	Y
C18	29	Female	Healthy	N	N	N	11.8	N	-	NT	NT	Y	Y
C19	46	Female	Healthy	N	N	N	49.5	N	-	NT	NT	Y	Y
C20	32	Female	Healthy	N	N	N	24.6	N	-	NT	NT	Y	Y

ID (W/F/O/C)	Age (years)	Gender (Male/Female)	Diagnosis	Allergic Rhinitis	Allergic Asthma	Chronic Urticaria	Total IgE KU/L	culprit	Cofactor	sIgE* kUA/L	SPT#	TMT	Pooled sample
C21	32	Male	Healthy	N	N	N	56.9	N	-	NT	NT	Y	Y
C22	29	Male	Healthy	N	N	N	8.0	N	-	NT	NT	Y	Y
C23	29	Male	Healthy	N	N	N	7.3	N	-	NT	NT	Y	Y
C24	34	Male	Healthy	N	N	N	14.0	N	-	NT	NT	Y	Y
C25	37	Male	Healthy	N	N	N	16.6	N	-	NT	NT	Y	Y
C26	34	Male	Healthy	N	N	N	2.0	N	-	NT	NT	Y	Y
C27	37	Male	Healthy	N	N	N	16.8	N	-	NT	NT	Y	Y
C28	39	Male	Healthy	N	N	N	15.5	N	-	NT	NT	Y	Y
C29	30	Male	Healthy	N	N	N	25.4	N	-	NT	NT	Y	Y
C30	48	Male	Healthy	N	N	N	12.1	N	-	NT	NT	Y	Y
C31	33	Male	Healthy	N	N	N	28.6	N	-	NT	NT	Y	Y
C32	36	Male	Healthy	N	N	N	9.6	N	-	NT	NT	Y	Y
C33	28	Male	Healthy	N	N	N	10.4	N	-	NT	NT	Y	Y
C34	27	Male	Healthy	N	N	N	14.6	N	-	NT	NT	Y	Y
C35	46	Male	Healthy	N	N	N	7.5	N	-	NT	NT	Y	Y
C36	45	Male	Healthy	N	N	N	19.6	N	-	NT	NT	Y	Y
C37	60	Male	Healthy	N	N	N	15.9	N	-	NT	NT	Y	Y
C38	47	Female	Healthy	N	N	N	13.8	N	-	NT	NT	Y	Y
C39	67	Female	Healthy	N	N	N	15.2	N	-	NT	NT	Y	Y
C40	62	Female	Healthy	N	N	N	8.7	N	-	NT	NT	Y	Y
C41	45	Female	Healthy	N	N	N	14.7	N	-	NT	NT	Y	Y

ID (W/F/O/C)	Age (years)	Gender (Male/Female)	Diagnosis	Allergic Rhinitis	Allergic Asthma	Chronic Urticaria	Total IgE KU/L	culprit	Cofactor	sIgE[*] kUA/L	SPT[#]	TMT	Pooled sample
C42	42	Female	Healthy	N	N	N	19.6	N	-	NT	NT	Y	Y
C43	27	Female	Healthy	N	N	N	58.6	N	-	NT	NT	Y	Y
C44	41	Female	Healthy	N	N	N	20.1	N	-	NT	NT	Y	Y
C45	31	Female	Healthy	N	N	N	43.9	N	-	NT	NT	Y	Y
C46	27	Female	Healthy	N	N	N	35.2	N	-	NT	NT	Y	Y
C47	30	Female	Healthy	N	N	N	54.7	N	-	NT	NT	Y	Y
C48	52	Male	Healthy	N	N	N	16.3	N	-	NT	NT	Y	Y
C49	27	Female	Healthy	N	N	N	41.0	N	-	NT	NT	Y	Y
C50	30	Male	Healthy	N	N	N	23.7	N	-	NT	NT	Y	Y
C51	27	Male	Healthy	N	N	N	50.8	N	-	NT	NT	Y	Y
C52	36	Male	Healthy	N	N	N	32.4	N	-	NT	NT	Y	Y
C53	38	Male	Healthy	N	N	N	27.9	N	-	NT	NT	Y	Y
C54	37	Female	Healthy	N	N	N	45.6	N	-	NT	NT	Y	Y
C55	33	Male	Healthy	N	N	N	39.1	N	-	NT	NT	Y	Y
C56	34	Female	Healthy	N	N	N	23.5	N	-	NT	NT	Y	Y
C57	29	Female	Healthy	N	N	N	34.8	N	-	NT	NT	N	N
C58	28	Female	Healthy	N	N	N	59.2	N	-	NT	NT	N	N
C59	30	Male	Healthy	N	N	N	14.5	N	-	NT	NT	N	N
C60	40	Male	Healthy	N	N	N	25.3	N	-	NT	NT	N	N
C61	43	Male	Healthy	N	N	N	47.4	N	-	NT	NT	N	N
C62	33	Male	Healthy	N	N	N	30.7	N	-	NT	NT	N	N
C63	28	Female	Healthy	N	N	N	53.7	N	-	NT	NT	N	N

ID (W/F/O/C)	Age (years)	Gender (Male/Female)	Diagnosis	Allergic Rhinitis	Allergic Asthma	Chronic Urticaria	Total IgE KU/L	culprit	Cofactor	sIgE* kUA/L	SPT#	TMT	Pooled sample
C64	35	Male	Healthy	N	N	N	19.1	N	-	NT	NT	N	N
C65	25	Female	Healthy	N	N	N	44.5	N	-	NT	NT	N	N
C66	58	Male	Healthy	N	N	N	37.5	N	-	NT	NT	N	N
C67	32	Female	Healthy	N	N	N	15.8	N	-	NT	NT	N	N
C68	48	Female	Healthy	N	N	N	54.3	N	-	NT	NT	N	N

***sIgE testing**: Specific IgE levels were measured using the ImmunoCAP™ system (Thermo Fisher Scientific, Sweden). Results are reported in kUA/L.

#**SPT methodology**: Skin prick testing was performed using food allergen extracts (Allergen Manufacturing and Research Center, Peking Union Medical College Hospital, Beijing, China). Histamine (5 mg/mL) and physiological saline served as positive and negative controls, respectively. A wheal diameter ⩾3 mm larger than the negative control at 15 minutes was considered positive.

Abbreviations: W, WDEIA patients; F, FIA patients; O, OAS patients; C, Healthy control. ID, Identity number; WDEIA, Wheat-dependent exercise-induced anaphylaxis; FIA, Food-induced anaphylaxis; OAS, Oral allergy syndrome; SPT, Skin prick test; TMT, Tandem mass tag-based quantitative mass spectrometry; N, No; Y, Yes; NT, Not tested.
